# Application of Sensory Descriptive Analysis and Consumer Studies to Investigate Traditional and Authentic Foods: A Review

**DOI:** 10.3390/foods8020054

**Published:** 2019-02-02

**Authors:** Jiyun Yang, Jeehyun Lee

**Affiliations:** Department of Food Science and Nutrition, Pusan National University, Busan 26241, Korea; jiyunyang@pusan.ac.kr

**Keywords:** sensory evaluation, specialty food, unique food products, ethnic food, descriptive analysis, consumer test

## Abstract

As globalization progresses, consumers are readily exposed to many foods from various cultures. The need for studying specialty and unique food products, sometimes known as traditional, authentic, ethnic, exotic, or artisanal foods, is increasing to accommodate consumers’ growing demands. However, the number of studies conducted on these types of products with good quality sensory testing is limited. In this review, we analyzed and reviewed sensory and consumer research on specialty and unique food products. Various factors such as manufacturing, processing, or preparation methods of the samples influence the characteristics of food products and their acceptability. Sensory descriptive analysis can be used to distinguish characteristics that highlight these differences, and consumer research is used to identify factors that affect acceptability. Familiarity with product attributes contributes to consumer acceptance. When cross-cultural consumer research is conducted to support product market placement and expansion, sensory descriptive analysis should be conducted in parallel to define product characteristics. This allows better prediction of descriptors that influence consumer acceptability, leading to appropriate product modification and successful introduction.

## 1. Introduction

In the field of food science, sensory science constitutes a discipline dealing with human sensory perceptions and affective responses to various kinds of foods, beverages, and their components that evolved from the need for scientifically sound and systematic sensory evaluation [1]. The conception of sensory science has been attributed to the development of consumer or hedonic food acceptance methodologies that were established in the 1940s by the U.S. Army Corps of Engineers [2]. More recently, sensory science has been defined as “a scientific method used to evoke, measure, analyze, and interpret those responses to products as perceived through the senses of sight, smell, touch, taste and hearing” [3]. Depending on the subject of the sensory science research, various methods may be used; among the sensory evaluation methods, sensory descriptive analysis and consumer acceptability testing are the most frequently used.

Sensory descriptive analysis involves the discrimination and description of both qualitative and quantitative sensory factors of products by trained panels [4,5]. For example, the Flavor Profile [6], Texture Profile [7], Spectrum^TM^ method [4], and Quantitative Descriptive Analysis (QDA^®^) [8] can be applied as sensory descriptive analysis methods. By using these kinds of methods, it is possible to pinpoint differences among product variants, conditions, identify drivers of consumer hedonic responses, and examine relationships between sensory and chemical characteristics [1,9].

Numerous studies related to the sensory descriptive analysis of food products or sensory methodologies have been published and the use of sensory methods related to product research and development has been described [10]. However, the number of papers reporting the results of good quality sensory descriptive analysis of ethnic, specialty, and exotic food products reflecting traditional and authentic food cultures is limited. The concept of “traditional” food is that of foods that represent a group of people, knowledge, and even local resources [11]. This uniqueness often is referred to as “authenticity”, which may be more often used for cultural products, rather than those that are part of our daily routines [12]. Food from different regions or countries can provide “unique” and “exotic” characteristics to individuals from other cultural backgrounds [13]. This often is regarded as “ethnic” foods outside the place of origin and when it is unfamiliar to specific individuals [11,14]. Because of the various possible definitions, in this review we have used the words “traditional”, “authentic”, “ethnic”, “exotic”, “unique”, and “specialty” interchangeably. 

Ethnic food can be defined narrowly as foods originating from a heritage and culture of an ethnic group who use their knowledge of local ingredients. More broadly, ethnic or traditional foods are representative of a cuisine of an ethnic group or country that is culturally and socially distinct and whose foods may be accepted by consumers outside of the respective ethnic group [15]. Ethnic foods provide consumers from other culinary traditions with opportunities to experience new cultures and cuisines [16]. For example, green tea [17,18], rooibos tea [19], fermented soybean [20,21], kimchi [22,23], Portuguese cooked blood sausage [24], and turrón [25] are kinds of ethnic foods that have been analyzed by researchers using sensory descriptive analysis for understanding their attributes.

Consumer acceptability represents one of the most important tests for sensory analysis and often involves a scaling method to measure the degree of liking or disliking of products using naive consumers [3]. However, the degree of acceptability does not constitute the only aspect of consumer studies [26]; consumer emotions, perception, and the relationship between consumers’ feelings about a product and descriptive sensory characteristics and instrumental information can also be determined [27]. Among those, food acceptance constitutes an essential outcome of the interaction between humans and foods [28]. Food acceptance may be affected by food habits, attitude, and beliefs [29,30], with culture (i.e., tradition) serving as one primary factor that underlies food choices [31,32]. Differences in the food environment and dietary experience across cultures may influence the preference for sensory characteristics of food products [33]. Similarly, familiarity with food products also may affect food choice [34] and food beliefs and potential acceptability [35]. Numerous consumer studies have been conducted on the acceptability of commercial food products as well as categories of food and various consumer methods, perceptions, emotions, and cross-cultural studies [36,37,38,39,40]. Although less prevalent, some consumer studies have also investigated traditional or unique food products such as traditional cheese [41,42,43,44,45], Doenjang (Korean traditional fermented soybean paste) [46,47], açai juice [48], polenta sticks [49], Argentinean fermented sausages [50], olive oil [51,52], and Korean traditional soup [53].

As globalization progresses, it has become easier to participate in and appreciate other cultures and many cultural traditions are being shared. This has led to an increasing number of food-savvy consumers and the trend for once-unfamiliar cuisines and flavors to become basic and standard elements in day-to-day diets of other cultures [54]. In particular, younger consumers are already accustomed to certain food products that may have been regarded previously as unique or novel. For example, concomitant with the increased exposure to ethnic or traditional foods, consumers enthusiastically use various kinds of spices to enhance the flavor of their dishes. In particular, in the U.S. market, spice consumption has grown almost three times as fast as the population over the last few decades [54,55]. The importance of not only spices however also other unique or special food products is growing in accordance with globalization and consumer needs. However, many more such foods exist than those that have already attained general awareness.

It is necessary to characterize these unique international food products to better understand and accommodate consumer demands for unfamiliar food products. In particular, no reviews have been published on sensory studies regarding such food products. The aim of the present review is, therefore, to describe and summarize the high-quality descriptive studies and consumer research that is currently available for special and unique food products such as traditional or ethnic foods. Accordingly, we will compare the conducted research that is based on the following perspectives: sample type and number and methods or procedure of descriptive and consumer studies. Data analysis and results will also be briefly compared between studies.

## 2. Literature Review

Articles were searched from the web-library of Pusan National University (https://lib.pusan.ac.kr) using keywords. In the case of sensory descriptive analysis, “descriptive sensory lexicon”, “descriptive sensory terminology”, and “descriptive sensory characteristics” were searched from 2000 to 15 August 2018. Consumer studies were searched using “consumer liking” for the same time period. Hundreds of papers were searched because of the wide range of keywords, although only a limited number of these evaluated traditional or ethnic foods as samples. All selected studies comprised research papers written in English and did not include any textbooks or papers written in different languages.

## 3. Sensory Descriptive Analysis

A total of 34 studies were reviewed for sensory descriptive analysis research using special food products. Table 1 shows the categories of the samples that were used and the corresponding reference articles. Food samples could be categorized into three broad groups of (a) beverages, (b) sauces, pastes, and dressings, and (c) a group of miscellaneous 20 other specific traditional food items that were difficult to categorize. Each category is discussed in detail in the following sections.

### 3.1. Beverages

In the beverage category, descriptive analyses of rooibos and green tea were included. Although the tea market is growing, we considered that rooibos and green tea are still classified as ethnic foods compared to black tea. A sensory wheel for rooibos was developed by Koch et al. [19]. Those authors used a total of 69 samples evaluated by nine panelists with extensive experience on descriptive analysis. They first developed 121 descriptors during the training sessions and then 27 terms were selected for inclusion in the sensory wheel based on their relevance. After testing, 17 attributes were eventually selected for efficient sensory profiling by grouping and eliminating descriptors. The suggested application of the sensory wheel was for use for the quality control of rooibos tea. For full profiling of rooibos tea, more descriptors might be needed [18]. Jolley et al. [56] also evaluated rooibos tea; samples were compared to determine differences based on production area and harvest year. Approximately nine to 10 trained female panelists, most of whom had previous experience on rooibos evaluation, participated and a total of 208 tea samples were assessed using the 17 characteristics developed by Koch et al. [19]. Understanding the results of the study was suggested as helping to understand product segments, opening up the opportunity for marketing niche products especially at the global level.

In the case of green tea, a greater number of studies have been conducted, likely reflecting increased awareness and consumption by global consumers worldwide. Lee and Chambers [17] examined differences among green teas from different countries and the correlation with consumer data using six panelists performing a sensory descriptive analysis on six samples using 18 attributes. These findings have the potential to explain the differences among samples from different production areas, processing methods, or with different flavor characteristics. Lee and Chambers [18] developed the lexicon of green tea from nine countries (China, India, Japan, Kenya, Korea, Sri Lanka, Taiwan, Tanzania, and Vietnam) and established definitions and references. They used a total of 138 green tea samples to generate descriptors that could distinguish all kinds of green tea samples. Specifically, 31 attributes were generated by six highly trained panelists who had more than 120 h of general descriptive training and averaged more than 1200 h of sensory descriptive testing. They used the flavor profile method, which usually involves a smaller number of panelists compared to other sensory descriptive methods. The consensus procedure was used however the original flavor profile method was modified by using a 0–15 scale. Subsequently, those authors further analyzed the data to cluster green teas based on their flavor profiles and found that the origin influenced flavors through a combination of varietal differences, growing conditions, and processing variations [57]. They noted that their study could be used as a marketing tool for consumer segments for green tea so that consumers would be able to select teas that met their specific sensory preferences. Those authors also identified flavor change during storage using two green tea samples with five different storage durations [58], suggesting that the tea retailer must consider the type of packaging of green tea in order to maintain its quality during the distribution period. In addition, other research groups also worked on green tea. Lee et al. [59] developed a method to establish sample preparation and presentation procedures using six green tea samples (three green teas × two grades) and evaluating 16 characteristics. They were attempting to create a method that would minimize possible bias caused by the changes in brewing temperature that could result in differences in volatile compounds of the tea. Those authors then analyzed differences in sensory attributes such as “turbidity” or “bitter taste” between green teas that were processed under different methods and the correlation of those sensory attributes with consumer acceptability [60]. They further evaluated decaffeinated green teas as samples to ascertain the product market of decaffeinated beverages potential [61].

### 3.2. Sauce, Paste and Dressing

In this category, soy sauce, Eshabwe (Ghee sauce), Gochujang (Korean chili paste), soybean paste, and Danish honey were included for review. In the case of sauce or paste, different characteristics were evaluated in different countries depending on the characteristics of the product. It is likely that the foods within this category may reflect specific food cultures of each country.

Soy sauce has become a widely used sauce originating from Asian countries. Jeong et al. [62] developed a lexicon with 22 attributes for understanding and establishing a standardized descriptive analysis procedure and descriptors for fermented soy sauce in various conditions. However, that study had limited samples. Thus, the developed lexicon only had eight descriptors that were similar to those found in a later study by Cherdchu et al. [63], which was based on a larger number of samples. Those attributes were alcohol, caramel, chemical, fermented, metallic, pungent, salty, and sour. Cultural differences, sample composition, or a difference in the range of samples chosen may be the reason for the difference in the lexicon. The article by Cherdchu et al. [63] developed 58 attributes using a wide range of 20 kinds of soy sauce (selected after screening an initial set of over 120 samples). The study included the participation of panelists from Thailand and the U.S. in this cross-cultural research project. In particular, they mentioned that language and culture constituted factors that limited the ability to describe certain characteristics, although they found ways to adapt to language issues by emphasizing the importance of using standard references to conduct well-communicated evaluations in cross-cultural studies. Imamura [64] later conducted a study of 149 mostly Japanese soy sauce samples that established 88 sensory descriptors, of which many were the same descriptors as those provided by Cherdchu et al. [63]. Imamura’s descriptors were divided into nine subgroups and were evaluated by 13–17 female descriptive panelists in each subgroup. A flavor wheel of soy sauce was developed to facilitate sensory evaluation and communication regarding sample qualities. In a follow-up study by Pujchakarn et al. [65], 9 female panelists, aged 38 to 56 years, developed 34 descriptors for seasoning soy sauce, a specific category of soy sauce, with the intent to provide new terms/references to add to prior soy sauce research. Soy sauce also was used as a sample for the investigation of the effects of different types of carriers [66]. That study showed changes in the flavor of soy sauce when used with differences in carriers such as rice, soup broth, and meat.

Eshabwe (Ghee sauce) comprises a traditional salty pudding-like condiment prepared from ghee or butter in western Uganda, eastern Congo, Rwanda, and Burundi [67]. Mukisa and Kiwanuka [67] established 15 characteristics with 10 panelists and evaluated the quality of samples. The developed descriptors consisted of not only flavor however also quality attributes such as soggy, musty, and stale. They considered that standardization of the food made following traditional processes crucial. Their results may be helpful for manufacturers to standardize Eshabwe processing methods to ensure product consistency.

Gochujang (Korean chili paste) was evaluated by Kim et al. [68], who reported the development of 34 attributes by 10 panelists for 31 different samples. Their aim was to investigate the characteristics of different Gochujang made by various producers and to determine any correlation with consumer age segments differing. Gochujang dressing, a similar product, was studied by Hong et al. [69], where eight panelists developed 10 sensory terms; in addition, cross-cultural consumer tests also were conducted in Korea, China, and the U.S. The terminology of their study was used to find drivers of consumer preference and it was mentioned that an understanding of the flavors of traditional foods was important for this purpose.

Soybean paste is a well-known food in Eastern Asia. Jung et al. [20] described 18 characteristics of 14 samples for comparing data from a panel performing a sensory descriptive analysis and electronic devices such as e-nose and e-tongue. From their findings, they emphasized the importance of sensory testing for describing a complex food matrix. Kim et al. [70] examined 30 sensory attributes of various soybean paste products available in Korean markets made with different manufacturing processes and then compared these with consumer acceptability data. They indicated that consumer acceptability was significantly influenced by sweet and monosodium glutamate (MSG) flavor characteristics of products. Chung and Chung [21] previously performed cross-cultural descriptive studies from which they developed 48 terms. Notably, they mentioned that the usage pattern of the same attribute differed cross-culturally; thus, further research was suggested to overcome the linguistic differences.

In total, 27 sensory descriptors of 21 Danish honeys were created using sensory descriptive analysis [71] with the aim to describe and differentiate the uniqueness of locally produced honeys in Denmark. Ten sensory panelists, six women and four men aged 20 to 62 participated in the evaluation. Their results might be used to help communicate the specific sensory quality of honeys; however, it would be difficult to promote local uniqueness, as they did not compare the Danish honey with honeys from other countries.

### 3.3. Miscellaneous Products

We also identified reports on 20 food products that could not be readily categorized together (Table 1). Tofu is a typical soybean product that constitutes one of the most favored ingredients of many East Asian style cuisines [72]. Chung et al. [72] developed 27 terms for various types of tofu, concluding that the sensory characteristics of tofu varied depending on numerous factors such as brand, processing method, and ingredients. Descriptors included ones related to appearance, odor/aroma, flavor/taste, texture/mouthfeel, and aftertaste categories. Moreover, Kamizake et al. [73] showed the effect of aging of soybean under different conditions on tofu quality. The tofu samples were made using two cultivars at three different conditions of soybean aging: control, accelerated aging, and natural aging. Those authors developed 16 sensory descriptors and found that the aging of soybean affected the sensory quality of tofu such as color, flavor, and texture attributes. However, all descriptors were highly expressed in principal component 1, meaning that most terms were correlated with each other in that study. In this study, 13 panelists participated, however information regarding their age, gender, and extent of training was not included. The high correlation among attributes may suggest that the panelists were not as well trained as those in the study by Chung (72). Between the two tofu studies [72,73], odor and flavor descriptors were mostly different probably because of differences in their purpose and samples and perhaps the training and experience of panelists. However, texture attributes were comparable although different descriptors were used such as springiness, hardness, easy to cut, and stickiness [72] compared to elasticity, firmness, fracturability, and residual adherence [73], respectively.

A lexicon for baechu kimchi was developed by Chambers et al. [22]. Prior to their research, little had been published regarding this side dish/condiment despite the wide variety of available kimchi preparations and processing methods that could affect kimchi quality. Because kimchi represents a highly variable food, those authors mentioned that future research should include studies of the sensory properties of kimchi made from various ingredients. Dongchimi, another type of kimchi that is made from radish with water, was examined by Cho et al. [23]. They analyzed the relationships between sensory data from descriptive analysis and chemical properties from instrumental analysis by partial least squares regression analysis (PLSR). Further research was suggested to facilitate Dongchimi product development. Although the main ingredients differed as baechu and radish were used, respectively [22,23], the nature of kimchi utilizing similar ingredients resulted in common descriptors such as sweet, salty, sour, garlic, red pepper (chili), fermented (yeast), green, heat burn (hot), and carbonation.

Certain meat products specific to certain regions have also been evaluated using sensory descriptive analysis. Pereira et al. [24] established 14 attributes for Portuguese cooked blood sausage using 12 products. Their results may help describe the sensory qualities of the blood sausages from different producers. Blood sausage variants are found worldwide [74,75,76], however sensory descriptive research on blood sausage from different cultures or countries were not conducted similarly enough to compare studies. Slovenian Krvavica was evaluated to initiate protected geographical indication; however, they used a 7–point scale that was somewhat similar to a just-about-right (JAR) scale for various attributes with 4 being an optimal intensity [74]. The sensory profile of commercial Spanish dry-cured sausage was evaluated by Ruiz Pérez-Cacho et al. [77], who commented that the developed terms might define and reveal differences among samples. Marangoni and Moura [78] developed 12 sensory terms for four different treatments of Italian salami to establish their sensory profile. Jo et al. [79] determined the sensory characteristics of Bulgogi, a Korean cooked meat dish. They also conducted cross-cultural consumer acceptability testing and compared these findings to the descriptive profile to understand the preference tendencies of different cultural consumers in order to investigate the market potential abroad.

A wide range of traditional grain-based foods have been tested. Italian polenta produced with 12 different corn cultivars during 2 years of testing was studied by Zeppa et al. [80], who defined 13 descriptors for the sensory profile of traditional polenta. Nine cultivars were examined in the first year and seven in the second year. Four cultivars were examined in both years. The 13 descriptors they selected were determined to have the maximum potential to identify attributes of each sample. They indicated that the defined lexicon might be broadly applied to describe the sensory qualities of polenta, in selecting cultivars of the base ingredients, and for use in product development. Doda burfi, an Indian milk cake, from different cities was characterized in order to standardize the product for globalization and widespread marketing of traditional items by Chawla et al. [81]. In that study, a panel established 19 characteristics that could explain differences among samples. Kim et al. [82] characterized Gangjung (a traditional Korean fried puffed snack) made via different treatments using 21 attributes. They suggested that the developed descriptors might be used for both characterizing samples and explaining the effect of sample preparation.

A lexicon of turrón, a nougat product that has European protected designation of origin (PDO) status, was defined by Vázquez-Araújo et al. [25] for various brands and commercial categories. A total of 41 sensory terms were evaluated by a trained panel in the U.S. and then the terms and definitions were translated and applied for use by native Spanish turrón quality panels. Those authors indicated that generally, the lexicon was successfully implemented, however that more studies should be conducted to overcome language and cultural/scientific differences (e.g., trained panels vs. quality panelists). They noted that reference product availability could be a potential barrier for such international work because they are not always available internationally. The sensory characteristics of dates were examined by Al-Farsi et al. [83]. They developed nine sensory attributes that were compared to compositional or nutritional components to help in the promotion of dates as a healthy food.

## 4. Consumer Studies

A total of 24 journal articles were reviewed for the consumer testing of special and unique food products. Table 2 shows the food samples that were used in each study, which were broadly categorized for the descriptive analysis as follows: beverages, sauce/paste/dressing, and miscellaneous other specific food products.

### 4.1. Beverages

Two different kinds of drinks accounted for products in the beverage category: green tea and açai juice. For green tea, Lee and Chambers [17] conducted consumer testing with U.S. consumers to examine how consumers liked green tea samples from three different countries and which characteristics were related to consumer preference. They analyzed their sensory descriptive and consumer data by correlation analysis and concluded that the relationship between flavor and liking was, as expected, complicated. Lee et al. [84] determined the relationship between acceptability ratings by consumers from three different countries (Korea, Thailand, and the U.S.) and flavor characteristics of green tea samples. They found that consumers who were familiar with green tea liked green vegetable attributes, however that the other group of consumers preferred green tea which did not have strong green tea flavor and bitterness. Because the acceptance of certain flavors was found to differ from one country to another (and within countries), the authors suggested that their findings implied that different food consumption experiences likely affect food selection and acceptability. Some other researchers mentioned that food consumption frequency affected acceptability [85] and familiarity influenced consumers’ discrimination ability of products [86]. To investigate consumer acceptance of unfamiliar food samples, Sabbe et al. [48] used açai juices with different concentrations of fruit juice. They found that familiarity was highly associated with consumer acceptability and that further studies were required to analyze the effect of repeated exposures.

### 4.2. Sauce, Paste and Dressing

As with the descriptive analysis, Doenjang, Gochujang products, soy sauce, Bulgogi (sauce), and olive oils were included in this category. Doenjang is widely used in Korea to make soup, sauce, or as a condiment. To determine consumer acceptance of the product and the underlying sensory drivers, Roh et al. [46] ascertained consumer preferences and associated factors such as product form. They showed that familiar food forms usually were consumed in practice. Another study determined consumer perception of Doenjang made by different manufacturing methods [47]. Also, Kim et al. [70] classified consumers into selected groups with similar preference patterns, finding that acceptability differed according to consumer segment. To understand consumer preference and the perception of Doenjang products, they recruited over 150 consumers to participate in each study. The larger number of consumer participants was used to expand the resulting understanding of consumer preferences. Similarly, some researchers [70,87] recruited more than 200 consumers to analyze the differences of liking between different age groups. The necessary number of consumers in sensory acceptability studies was calculated as approximately 100 or more for each segment that was compared [88].

Gochujang products have also been evaluated for consumer acceptability and to better understand their acceptance. The sensory drivers of positive response in different age groups were investigated by Kim et al. [68]. Hong et al. [69] conducted cross-cultural consumer testing between Korea, China, and the U.S. to provide information on country-specific factors affecting consumer acceptance of Gochujang dressing with different ratios of each ingredient. Because the aims of both studies were to investigate consumer acceptance factors for those products, descriptive analyses were conducted first to identify sensory characteristics of the food samples. Although the samples differed in these two studies, the sweet characteristic tended to influence Korean consumers positively in both studies.

Studies also have used soy sauce and Bulgogi together as samples to evaluate consumer acceptance because soy sauce is considered a basic ingredient for Bulgogi marinade sauce. Park et al. [89] conducted a cross-cultural study between Korean and Japanese consumer sensory perception and hedonic responses, from which they suggested that cultural background affected sensory perception. They mentioned, as other authors suggested, that cultural background affected food experiences and familiarity which could moderate consumers’ sensory response to ethnic foods [53,79,90,91]. Heo and Lee [92] determined the acceptability of different brands of soy sauce and Bulgogi by U.S. consumers who were unfamiliar with the specific samples that were tested. They suggested a possible relationship between familiarity and liking because the soy sauce with familiar flavor had higher acceptability when tasted both in its original form and when used in cooking by consumers. Another study used both soy sauce and Bulgogi samples for comparing hedonic information between Korean and Chinese consumers [79]. Overall, familiarity or cultural differences appeared to influence the results of these cross-cultural studies. Korean consumers were positively influenced by the sweet taste of Bulgogi, however the opposite was true for Chinese consumers. The authors explained this finding by explaining that Chinese meat dishes usually do not have much sweet flavor. The authors implied that it was important to understand the cultural context of consumers when evaluating or promoting ethnic foods and flavors.

Olive oil has been used widely as an ingredient or sauce in Western countries. Delgado et al. [52] studied consumer liking and perceptions of extra olive virgin oils by Northern California consumers based on product packaging and labeling. That study identified some discrepancies between the overall liking evaluations based on packaging versus flavor. In their study, the “packaging group” considered the region of origin as a key point and the “blind group” evaluated the olive oil based on sensory characteristics such as bitterness or pungency. They mentioned that local or national product reputation or expectations could be an important motivation for purchasing. Pagliuca and Scarpato [51] analyzed and compared preferences of Italian olive oils between generic consumers and experts. They indicated that experts emphasized the recognition of intrinsic attributes more than novices because only the experts recognized the differences between oils from PDO compared to non-PDO oils. The degree of awareness regarding the EU certification system and geographical indications may explain this differential. Another study [93] of six olive oils compared U.S. and Spanish consumers and found that Spanish consumers liked the more bitter and green flavors of extra virgin olive oils while U.S. consumers liked the more bland, fruity, or floral flavors of the other two oils. They concluded that Spanish consumers may be more familiar with various olive oils and, thus, appreciated different characteristics. Thus, unlike most other ethnic foods, studies of olive oil have been conducted to understand consumer perceptions of various aspects in addition to their acceptance of its flavor characteristics.

### 4.3. Miscellaneous Products

The category of other foods included some cheese products, tofu, meat products, and additional foods as shown in Table 2. Although cheese comprises a well-known food product worldwide, some traditional types, which are authentic to specific regions, still constitute special and unfamiliar foods to many consumers. Ojeda et al. [42] used Idiazabal cheese with three different quality levels to compare the liking of local consumers in Spain with the sensory quality as assessed by trained panelists. In that study, the preferences of a large group of consumers was mainly driven by certain characteristics such as “sweet” and “toasty” attributes described by the trained panel. Scamorza cheese with different milk and starter type was studied by Braghieri et al. [43] in Italy who identified the driver of liking in terms of specific sensory input. They mentioned that the liking of specific sensory attributes such as appearance, taste/flavor, and texture were highly related to overall liking. They concluded that further studies were needed to promote product differentiation and to meet the sensory requirements of particular consumer segments. Swiss cheeses including different kinds of retail products were evaluated in the U.S. by Liggett et al. [45], who investigated the relationship between consumer liking and the specific flavor characteristics. For establishing the characteristics of unique food products and understanding consumer acceptance, descriptive analysis may be combined with consumer testing to better understand specific foods.

The acceptance of tofu prepared using different soybean conditions was studied by Kamizake et al. [73]. It was found that the condition of soybeans could significantly impair tofu quality.

As with descriptive analysis, certain meat products of specific regions were used for consumer studies. Lucanian dry cured sausages were examined by Braghieri et al. [94] who found that both intrinsic and extrinsic aspects such as taste, animal health, and preservation affected consumer choice. López et al. [50] explained that consumer acceptance of Argentinean fermented sausages from different regions represented a quality factor that was as important as instrumental analysis to understand differences. These results suggested that consumer acceptability constitutes an important component of quality control.

The sensory characteristics of Thai dried chili prepared with different treatments were determined by Toontom et al. [95] along with consumer testing. Familiarity and positive experiences were suggested to be associated with consumer acceptance. However, to understand international consumer responses and target the global market, cross-cultural consumer studies are needed. To this end, Jang et al. [53] compared the consumer acceptance of different kinds of Korean traditional soup between U.S. and Japanese consumers. They suggested that strategic modification of food flavor might help decrease neophobic responses from target consumers. In turn, the dynamic overall preference of polenta sticks was studied by Di Monaco et al. [49], which indicated that both food properties and food consumption duration affected the preference for a product. Finally, Saleh et al. [96] examined the effect of filling on the acceptability of a Jordanian traditional dessert, which determined that the ratio of ingredients could affect product acceptability for consumers. In their study, the filling with a high date ratio mostly affected hardness of the sample and its acceptability.

## 5. Discussion

Numerous kinds of unique and special food products exist, however only a small number of those have been studied using good sensory methods. We summarized the sensory studies of traditional and authentic food products. There were research projects conducted for both sensory descriptive analysis and consumer study to develop attributes, analyze consumer preference/perception, and identify the factors affecting consumer acceptance. Both methods were important to understand food products and their consumer perception. Where only sensory descriptive analysis was conducted, researchers could understand the attributes and intensities of the sample and potentially differentiate among samples with different ingredients, processing, storage conditions, etc. However, researchers could not determine which attributes would be related to consumer acceptance. Similarly, studies that used only consumers could understand consumer preference, however they could not prove which attributes affected consumer acceptance and perception. For the deepest understanding of consumer products, both methods should be used.

Some limitations were observed in the studies. For descriptive analysis in this review, there were large variations in the numbers of panelists that were used. The minimum panelist number was 5 [22] and the maximum number of panelists was 18–19 [24,81]. It is not certain whether the difference in the number of panelists affects the reliability of the sensory results, however it is clear that there are differences between methods for each descriptive study. Actually, the recommended ideal number of judges was not clear. Although the number of panelists varies by method, researchers should provide a clear basis if their participant number was sufficient. Chambers et al. [97] pointed out that the number of panelists necessary for descriptive panels was dependent on the training of the assessors and the types of attributes that were evaluated, with fewer being needed when the training/experience level was higher or when the attributes were more easily evaluated.

There was also variation among the number of participants in consumer tests. Consumers who participated in consumer tests should be representative of potential consumers of the product [97]. Thus, generally 100 or more consumers need to be recruited per segment for quantitative consumer tests to be reliable. Because some of the consumer tests in this review were conducted with small numbers of consumers, it is important to understand that the reliability of those studies may be less than others. For example, Hong et al. [69] conducted a cross-cultural study of Gochujang dressing with Korean (*n* = 50), Chinese (*n* = 34), and U.S. (*n* = 26) citizens. Although the total number of consumers was more than 100, participants from each country were not enough to be representative of populations in each country. Other research [49,96] also had small numbers of consumers. Consumer tests should be conducted with sufficient numbers of consumers because otherwise they may present unreliable results that are not representative of the population. If conducting consumer studies with a smaller number of participants, researchers must justify their reasoning for deviating from recommendations.

## 6. Conclusions

In this review, we reviewed sensory analysis (i.e., evaluation) studies regarding traditional and authentic food products, which sometimes are called specialty, exotic, or unique foods. The purpose of our review was to provide a broad summary of the descriptive analyses and consumer research that is currently available for various traditional and authentic food products. As globalization progresses, the need for understanding flavors of traditional, unique, regional, or artisanal foods is clearly increasing in order to better comprehend and accommodate consumer demands for unfamiliar food products. Additional sensory science research using unusual and ethnic food products should be carried out to better understand product characteristics.

Numerous studies highlighted the effect of familiarity on consumer perception. In particular, a positive correlation between familiarity and consumer acceptance was reported frequently [91,97,98,99,100]. The consumers who were familiar with the sample showed higher liking than non-familiar consumers [91]. In addition, descriptive analysis could also be affected by familiarity. Drake et al. [101] noted that lexicon development could not be freed from the influence of the culture from which the panel originated, although other authors [25,63] have reported that cross-cultural studies using lexicons certainly are possible with planning and coordination.

To obtain deeper insights regarding specialty and unique food products, large numbers of studies using different kinds of traditional, unique, or ethnic food items and categories from a range of countries or regions or made from various ingredients or processing methods should be carried out. In addition, it is necessary to conduct cross-cultural descriptive and consumer studies to establish universal lexicons and to understand the attributes that influence consumers’ liking.

## Figures and Tables

**Table 1:** Sensory descriptive analysis studies using traditional foods as samples.

Food Category	Food Sample	Reference ^1^	Sample Number	Descriptive Panelist Number	Number of Characteristics
*Beverages*	Rooibos	Jolley, Van der Rijst, Joubert, & Muller [56];	69	9	17
		Koch, Muller, Joubert, Van der Rijst, & Næs [19]	208	9–10	17
	Green tea	Lee, Chambers, & Chambers [57];	138	6	32
		Lee & Chambers* [17];	6	6	18
		Lee & Chambers [58];	10	6	20
		Lee, Lee, Kim, & Kim* [61];	8	9	13
		Lee, Lee, Sung, Lee, & Kim* [60];	7	8	15
		Lee, Chung, Lee, Lee, Kim, & Kim [59];	6	8	16
		Lee & Chambers [18]	138	6	31
*Sauce paste and dressing*	Soy sauce	Imamura [64];	149	13–17	88
		Pujchakarn, Suwonsichon, & Suwonsichon [65];	925	9	34
		Cherdchu & Chambers IV [66];	20	6	87–89
		Cherdchu, Chambers IV, & Suwonsichon [63];	20	9/6	58
		Jeong, Chung, Suh, Suh, & Kim [62]	6	8	22
	Eshabwe (Ghee sauce)	Mukisa & Kiwanuka [67]	4	10	15
	Gochujang (Korean chili paste)	Kim, Go, Kim, & Chung* [68]	31	10	34
	Soybean paste	Jung et al. [20];	14	8	18
		Kim, Hong, Song, Shin, & Kim* [70];	7	8	30
		Chung & Chung [21]	8	10/9	48
	Gochujang dressing	Hong, Lee, Chung, Chung, Kim, & Kim* [69]	6	8	10
	Danish honey	Stolzenbach, Byrne, & Bredie [71]	21	10	27
*Miscellaneous Other Products*	Tofu	Kamizake, Silva, & Prudencio* [73];	6	13	16
		Chung, Lee, & Chung [72]	7	6	27
	Kimchi	Chambers, Lee, Chun, & Miller [22];	10	5	17
		Cho, Lee, Choi, & Chung [23]	9	10	22
	Portuguese cooked blood sausage	Pereira, Dionísio, Matos, & Patarata [24]	12	18	14
	Spanish dry-cured sausage	Pérez-Cacho, Galán-Soldevilla, Crespo, & Recio [77]	7	5	26
	Italian salami	Marangoni & Moura [78]	4	12	12
	Bulgogi	Jo, Lee, Sohn, & Kim* [79]	6	8	20
	Italian polenta	Zeppa, Bertolino, & Rolle [80]	12	10	27
	Doda burfi (Indian milk cake)	Chawla, Patil, & Singh [81]	16	19	19
	Gangjung (Korean oil-puffed snack)	Kim, Kim, Chung, Lee, & Kim [82]	10	8	21
	Turrón	Vázquez-Araújo, Chambers, & Carbonell-Barrachina [25]	67	6	41
	Date	Al-Farsi, Alasalvar, Morris, Baron, & Shahidi [83]	3	10	9

^1^ Asterisk (*) indicates that the study was conducted with consumer testing.

**Table 2:** Consumer acceptability studies using traditional foods as samples.

Food Category	Food Sample	Reference ^1^	Sample Number	Consumer Number	Country
*Beverages*	Green tea	Lee & Chambers, 2010a* [17];	12	410	USA
		Lee, Chambers IV, Chambers, Chun, Oupadissakoon, & Johnson [84]	6	120/239/100	Korea/Thailand/USA
	Açai juice	Sabbe, Verbeke, Deliza, Matta, & Van Damme [48]	6	123	Belgium
*Sauce, paste and dressing*	Doenjang (soybean paste)	Roh, Lee, Kim, & Kim* [46];	8	157	Korea
		Kim & Lee [47];	4	291	Korea
		Kim, Hong, Song, Shin, & Kim* [70];	7	200	Korea
	Gochujang (Korean chilli paste)	Kim, Go, Kim, & Chung* [68]	4	280	Korea
	Gochujang dressing	Hong, Lee, Chung, Chung, Kim, & Kim* [69]	6	50/34/26	Korea/China/USA ^2^
	Bulgogi marinade sauces	Park, Ko, Jang, & Hong [89]	7	102/100	Korea/Japan
	Soy sauce, bulgogi	Heo & Lee, 2017 [92]	4	123	USA
		Jo, Lee, Sohn, & Kim [79]	6	76/120(72)	Korea/China
	Olive oil	Delgado, Gómez-Rico, & Guinard [52]	18	212	USA
		Pagliuca & Scarpato* [51]	5	400/35	Italy
		Vázquez-Araújo, Adhikari, Chambers, Chambers, & Carbonell-Barrachina* [93]	6	100/100	Spain/USA
*Others*	Idiazabal cheese	Ojeda, Etaio, Guerrero, Fernández-Gil, & Pérez-Elortondo* [42]	9	212	Spain
	Scamorza cheese	Braghieri, Piazzolla, Romaniello, Paladino, Ricciardi, & Napolitano* [43]	4	87	Italy
	Swiss cheese	Liggett, Drake, & Delwiche* [45]	15	101	USA
	Lucanian dry cured sausages	Braghieri, Piazzolla, Carlucci, Bragaglio, & Napolitano* [94]	10	102	Italy
	Argentinean Fermented Sausages	López, Bru, Vignolo, & Fadda [50]	10	120	Argentina
	Thai dried chili	Toontom, Posri, Lertsiri, & Meenune* [95]	4	120	Thailand
	Traditional Korean soup	Jang, Hong & Kim [53]	6	104/106/100	USA, Japan
	Polenta stick	Di Monaco, Miele, Volpe, Masi, & Cavella [49]	4	50	Italy
	Traditional Jordanian dessert	Saleh, Akash, Al-Dabbas, & Al-Ismail [96]	6	40	Jordan

^1^ Asterisk (*) indicates that the study was conducted with sensory descriptive analysis. ^2^ Consumer test was conducted in Seoul, Korea, however it included consumers of each nationality.
